# Does the number of previous mood episodes moderate the relationship between alcohol use, smoking and mood in bipolar outpatients?

**DOI:** 10.1186/s12888-017-1341-z

**Published:** 2017-05-15

**Authors:** Wendela G. ter Meulen, Jan van Zaane, Stasja Draisma, Aartjan T.F. Beekman, Ralph W. Kupka

**Affiliations:** GGZ inGeest and Department of Psychiatry, Amsterdam Public Health research institute, VU University Medical Center, A.J. Ernststraat 1187, 1081 HL Amsterdam, The Netherlands

**Keywords:** Kindling, Alcohol, Smoking, Bipolar disorders

## Abstract

**Background:**

Evidence suggests that alcohol use and smoking are negatively associated with mood in bipolar disorders (BD). It is unknown if this relationship is moderated by the number of previous mood episodes. Therefore, this paper aims to examine whether the number of previous mood episodes moderates the relationship between alcohol use and smoking, and mood.

**Method:**

This study assessed the outcomes of 108 outpatients with BD I and II in a prospective observational cohort study. For 1 year, subjects daily registered mood symptoms and substance use with the prospective Life Chart Method. The relationship between the average daily consumption of alcohol and tobacco units in the whole year and mood were examined by multiple linear regression analyses. Number of previous mood episodes, grouped into its quartiles, was added as effect moderator. Outcome was the number of depressive, hypomanic and manic days in that year.

**Results:**

The number of depressive days in a year increased by 4% (adjusted β per unit tobacco = 1.040; 95% CI 1.003–1.079; *p* = 0.033) per unit increase in average daily tobacco consumption in that same year. Interaction analyses showed that in those subjects with less than 7 previous mood episodes, the number of manic and hypomanic days increased by 100.3% per unit increase in alcohol consumption (adjusted β per unit alcohol = 2.003; 95% CI 1.225–3.274; *p* = 0.006). In those with 7 to 13 previous mood episodes, the number of manic and hypomanic days decreased by 28.7% per unit increase in alcohol consumption (adjusted β per unit alcohol = 0.713; 95% CI 0.539–0.944; *p* = 0.019); and in subjects with 14 to 44 previous mood episodes, the number of manic and hypomanic days decreased by 7.2% per unit increase in tobacco consumption (adjusted β per unit tobacco = 0.928; 95% CI 0.871–0.989; *p* = 0.021).

**Conclusions:**

The number of previous mood episodes moderates the relationship between alcohol use and smoking and mood; and smoking is adversely associated with the number of depressive days.

## Background

Bipolar disorder (BD) is a relatively common mood disorder, with a lifetime prevalence of 1.3% in the adult Dutch population [1] and estimates of 1–3% worldwide [1]. On average, patients spend more time in depressive than in manic or hypomanic episodes [2]. Even when patients receive adequate treatment, the naturalistic course of BD is characterized by recurrences of manic, hypomanic and depressive episodes [3], that occur in up to 50% of BD patients within one year after recovery from a mood episode and in up to 90% of BD patients during their lifetime [4]. Therefore, it is relevant to identify those factors that impact on mood, as these factors may be a target for intervention.

Alcohol use and smoking are frequent co-travelers with BD [5–7] and a growing body of evidence suggests that these substances may adversely affect mood in subjects with BD. For example, alcohol use disorder (AUD) was associated with a worse presentation of acute mania, more affective episodes, increased mood recurrence and increased rapid cycling in cross-sectional studies (for an overview: see [7]). Prospective studies have reported an increased risk of depressive relapse and mood recurrence in those with AUD compared to those without AUD [8–10]. Likewise, smoking was associated with more rapid cycling, an increased frequency of depressive and manic episodes, and a higher symptom severity in cross-sectional studies [11, 12]. Prospective studies have demonstrated worse Clinical Global Impression BD (CGI-BD) scores and lengthier hospital stays [12]. Although explanations for the association between alcohol, smoking and mood are multidirectional [7, 13, 14], the adverse relationship between alcohol use and smoking, and mood may be mediated by neuropathogenic changes including neurochemical alterations and oxidative stress [13, 15, 16].

By contrast, a number of studies found no relationship between alcohol and smoking on the one hand, and mood on the other in BD [13, 17–19]. This inconclusiveness between studies may first be explained by methodological caveats, including few prospective studies, large heterogeneity in definitions of alcohol use and smoking, and divergent analytic approaches to investigate either shorter- or longer term effects. Furthermore, the relationship between alcohol use, smoking and mood may depend on the number of previous mood episodes: Post et al.’s kindling theory [20–22] states that the initial episodes of affective disorders are often precipitated by exogenous stressors whereas mood episodes tend to occur more autonomously after successive recurrences. In unipolar depression for example, the association between stressful life events and depression progressively declines with increasing episode number [20, 23]. Likewise, cognitive behavioral therapy (CBT) and pharmacological treatment are more effective in those BD subjects with few compared to multiple episodes [24–26]. Thus, alcohol and smoking may predominantly interact with mood in BD subjects with few compared to multiple previous mood episodes. One study [8] showed that AUD increases the risk of hospital re-admission for a new bipolar mood episode during the first episodes (measured as admissions), but not after multiple re-admissions.

To our knowledge, no research to date has investigated if such a “window of vulnerability” exists for the relationship between alcohol use, smoking and mood in BD subjects with fewer compared to multiple episodes. Therefore, this paper aims to examine whether the number of previous mood episodes moderates the association between the amount of alcohol use and smoking in one year, and the number of experienced depressive, hypomanic and manic days in that same year of follow up of daily life chart scores. We hypothesized that the amount of consumed alcohol and cigarettes is negatively associated with mood in BD, and that these associations are most pronounced in those subjects who have experienced fewer compared to multiple lifetime mood episodes.

## Methods

### Study sample and recruitment

We used the data of the Alcohol, Drugs and Stimulants (ADS) study of van Zaane et al. [18, 27] in a secondary analysis. This is a prospective study with the aim to investigate the effects of daily ADS intake on the course and outcome of patients with BD during a whole year through daily life chart recordings. The inclusion of subjects took place between June 2003 and November 2005. Exclusion criteria and assessment procedures are described in detail elsewhere [17]. Briefly, subjects were considered for enrolment in the ADS-study if they were aged 18–75 years and met criteria of BD I or II as assessed by the Structured Clinical Interview for DSM-IV (SCID-I; [28]). Outpatients were approached from mental hospitals, an addiction treatment center, and through the Dutch Association for Manic-Depressive Patients and Relatives. A total of 158 subjects met the inclusion criteria and completed baseline assessment. Out of these 158, a total of 108 subjects (68%) aged 23–68 completed daily life charts during the whole year. Reasons for the 50 non-completers were: aversion or non-adherence to the daily registrations (*n* = 22), too severe mood symptoms (*n* = 4), death due to liver coma (*n* = 1), daily registrations too confrontational (*n* = 4), and unknown reasons (*n* = 23). Compared to completers, non-completers were more often male; no other significant baseline sociodemographic or clinical differences were found.

### Ethics

The ADS study was approved by the Medical Ethical Review Committee of the University Medical Center Utrecht, The Netherlands. All subjects gave written informed consent after full explanation of the study.

### Assessments

Data were collected at baseline by a team of mental health care professionals who were trained to perform the SCID-I by RWK. The SCID-I assessment included number of previous mood episodes, history of rapid cycling, and comorbid diagnoses such as anxiety, alcohol and other substance use disorders. A baseline Clinical Global Impressions Scale-Bipolar Version (CGI-BD [28]) score was obtained. Additional data were obtained with the Network Enrollment Questionnaire (NEQ) of the former Stanley Foundation Bipolar Network [29]. The NEQ assesses demographic, social and clinical characteristics, including family history of psychiatric illness, and various details about alcohol consumption and smoking. For a period up to one year subjects daily completed the National Institute of Mental Health prospective Life Chart Methodology (NIMH-LCM-p, further referred to as LCM-p [30]). At baseline and at every monthly visit during follow up, the LCM registrations were checked and approved by the research assistants, who were psychologists with ample clinical experience. Subjects got written and verbal instructions to daily assess their mood symptoms and severity, the related level of dysfunction, medication use, consumption of alcohol, cigarettes and other substances with the LCM-p.

### Definition of outcome

The LCM-p allows measurement of the severity of mood symptoms and related level of dysfunction from both affective poles on a 5-point scale (0 = no, 2.5 = mild, 5 = low moderate, 7.5 = high moderate, and 10 = severe dysfunction). As previous studies showed inconsistent results regarding the relationship between alcohol use and smoking on the one hand and either depressive or (hypo) manic outcomes on the other hand, we assessed both affective poles separately. The outcome measures, based on the complete year of the study, therefore were: 1. number of depression days (LCM-p mood score per 1 day of at least low moderate depression); and 2. number of hypomania and mania days (LCM-p mood score per 1 day of at least mild mania).

### Definition of alcohol and smoking

Subjects daily scored their alcohol and tobacco consumption with the LCM-p. One unit of alcohol was defined as 12 mL pure alcohol equaling 100 mL of wine (12% alcohol), 250 mL of beer (5% alcohol), or 35 mL of liquor (35% alcohol), according to the Netherlands Institute for Mental Health (https://www.trimbos.nl). One tobacco product was defined as one cigarette, including self-rolled cigarettes from common tobacco, regardless of nicotine content. We calculated the average numbers of alcohol units and tobacco products per day based on the whole year of LCM-p registration.

### Confounders for the effects of alcohol and smoking

Confounders were chosen based on *a priori* hypotheses. Adjustments were made for age at intake in the ADS study, gender and socioeconomic status as measured by level of income [31–33]. Age at onset of BD was also considered a possible confounder [34–36]. Rapid cycling [37] was not entered as a confounder in the model due to collinearity with number of episodes.

### Effect modification: Number of mood episodes

The number of mood episodes that subjects had experienced in their lifetime was based on the SCID-I assessment, or on the NEQ if this variable was not available from the SCID-I (*n* = 1). To investigate effect modification, this variable was grouped into its quartiles that successively consisted of less than 7, 7 to 13, 14 to 44, and more than 44 mood episodes.

### Statistical analysis

The baseline sociodemographic and clinical characteristics of all BD subjects were summarized using descriptive statistics. To give a clinical impression of sample characteristics, bivariate differences were calculated for low and high number of episodes, based on the median number of mood episodes at baseline as cutoff, which in our sample coincided with 13 episodes. For categorical data, *p*-values were calculated with the Chi-square test. For the numerical data, unpaired t-tests (if normally distributed data) and Mann-Whitney U tests (if non-normally distributed data) were used for comparing between groups. For all tests, a *p*-value of <0.05 was regarded as statistically significant.

Because the numbers of hypomanic, manic and depressive days were positively skewed, we applied natural logarithmic (ln) transformation on these outcome scores, which enabled multiple linear regression analyses. This analytic approach was chosen to focus attention on the moderating effect of number of previous episodes on the relationship between alcohol and smoking and the number of experienced ill days (either depressed or (hypo)manic) during a whole year. The hypothesis that the average daily consumption of alcohol and cigarettes is negatively associated with mood in BD, was tested by multiple linear regression analyses with linear alcohol and smoking predictor variables, and without interaction terms. Adjusted beta’s (β) and their 95% confidence intervals (CIs) were calculated. The main hypothesis – that the associations between the amounts of alcohol use, smoking and mood are most pronounced in those subjects who have experienced fewer compared to multiple lifetime mood episodes – was tested by the addition of interaction terms to the regression models. These interaction terms were created by multiplying the quartiles of number of episodes *by* respectively the alcohol and smoking variables. The omnibus interaction term was considered significant at a *p*-value of <0.05.

Analyses were conducted using IBM SPSS Statistics version 22.0 (SPSS Inc., Chicago, USA). Confounders included in the models were selected by enter method and using a *p*-value of <0.05.

## Results

### Baseline characteristics

Sample characteristics, including sociodemographic and clinical characteristics, of the 108 patients are presented in Table 1. From this table it can be seen that the mean duration of illness was 22.3 years, and the median number of previous mood episodes 13.5 (IQR 7.0–46.3). Significant differences between those with multiple (>13) compared to fewer (≤13) episodes included a higher age at intake, more rapid cycling, and a greater duration of illness.

**Table 1:** Sample characteristics at baseline, and comparison between those with 13 or less episodes, versus those with more than 13 episodes (*n* = 108)

Sociodemographics	All 108 subjects	≤13 episodes**	>13 episodes**	*p*-value*
Gender (% female)	54.6	56.6	52.8	0.696
Age at intake (mean in years, SD)	46.1 (10.0)	43.5 (10.1)	48.6 (9.5)	0.008*
Educational level: % ≥ high school	51.4	45.3	59.6	0.141
Income (% >20.000 euro/year)	36.5	31.4	43.1	0.219
Unable to work (%)	50.0	50.9	49.1	0.846
Clinical characteristics				
BD I (% BD I; % BDII = 100-%BDI)	62.0	69.8	54.7	0.109
Liftetime history of rapid cycling (%)	17.0	5.8	26.9	0.004*
First degree relative with BD (%)	40.7	37.3	45.5	0.430
Age at onset of BD (mean in years, SD)	23.8 (9.7)	25.4 (10.3)	22.3 (8.7)	0.097
Duration of BD (mean in years, SD)	22.3 (11.7)	18.1 (10.8)	26.3 (10.9)	<0.001*
Current diagnosis alcohol dependence (%)	20.2	13.5	26.9	0.087
Current daily smoking (%)	41.7	43.4	39.6	0.693
Current diagnosis drug use/dependence (%)	3.7	1.9	5.7	0.308
Current diagnosis anxiety disorder (%)	15.7	11.3	18.9	0.278
Lifetime nr. of mood episodes (median, IQR)	13.5 (7.0–46.3)	7.0 (4.5–10.0)	45.0 (20.0–80.0)	<0.001*
Uses any medication in the last month (%)	92.5	90.4	94.2	0.462
Mean units of alcohol per day at baseline	2.1	2.0	2.2	0.652
Mean units of tobacco at baseline	7.3	7.6	6.8	0.722

*Bolded *p*-values denote significance.

**Differences between those with few compared to multiple episodes, based on the median number of episodes of 13

### Alcohol use and smoking

Alcohol consumption was not associated with more depressive or hypomanic and manic days for the whole year, after adjustment for confounders (adjusted β’s at a *p*-value of >0.05, see Table 2). The number of depressive days independently increased by 4.0% per unit increase in tobacco consumption per year (adjusted β per unit tobacco = 1.040; 95% CI 1.003–1.079; *p* = 0.033).

**Table 2:** Associations and interaction analyses for alcohol/smoking severity, and mood in BD in one-year follow-up (*n* = 108)

	Number of depressive days per year				Number of (hypo+)manic days per year			
	95% CI^b^				95% CI^b^			
	β^b^	Lower bound	Upper bound	*p*-value*	β^b^	Lower bound	Upper bound	*p*-value*
Alcohol use^a^	0.961	0.791	1.﻿﻿168	0.687	0.994	0.843	1.171	0.938
Interaction of alcohol use *by* quartile^a^:								
First quartile^c^	0.819	0.448	1.﻿496	0.512	2.003	1.225	3.274	**0.006**
Second quartile^c^	0.812	0.579	1.140	0.226	0.713	0.539	0.944	**0.019**
Third quartile^c^	1.004	0.708	1.426	0.980	0.885	0.659	1.﻿190	0.416
Fourth quartile^c^	1.267	0.922	1.741	0.142	1.﻿228	0.939	1.607	0.132
Smoking^a^	1﻿.040	1.003	1.079	**0.033**	0.982	0.952	1.﻿013	0.256
Interaction of smoking *by* quartile^a^:								
First quartile^c^	0.999	0.893	1﻿.118	0.988	1.064	0.967	1﻿.170	0.201
Second quartile^c^	0.994	0.914	1.080	0.878	1.021	0.952	1.095	0.553
Third quartile^c^	0.965	0.893	1.042	0.358	0.928	0.871	0.989	**0.021**
Fourth quartile^c^	1.043	0.952	1.142	0.365	1.052	0.976	1.135	0.182

^a^Average alcohol use and smoking per day based on the whole year, adjusted for gender, age at intake in the ADS study, socioeconomic status and age at onset of BD.

*Bolded *p*-values denote significance.

^b^Analyses over natural logarithmic transformed values; presented values are the back-transformed values.

^c^In each analysis, the effect of the mentioned quartile is compared to the other three quartiles as reference categories. Quartiles refer to <7, 7–13, 14–44 and >44 previous mood episodes, respectively

### Interaction analyses

Interaction analyses showed that the number of previous mood episodes moderated the associations between alcohol use and smoking, and number of days ill. First, in those subjects in the first quartile, with less than 7 previous mood episodes, one unit increase in alcohol consumption increased the number of manic and hypomanic days by 100.3% (adjusted β per unit alcohol = 2.003; 95% CI 1.225–3.274; *p* = 0.006). Second, in those subjects in the second quartile, with 7 to 13 previous mood episodes, one unit increase in alcohol consumption decreased the number of manic and hypomanic days by 28.7% (adjusted β per unit alcohol = 0.713; 95% CI 0.539–0.944; *p* = 0.019). Third, in those subjects in the third quartile, with 14 to 44 previous mood episodes, one unit increase in tobacco consumption decreased the number of manic and hypomanic days by 7.2% (adjusted β per unit tobacco = 0.928; 95% CI 0.871–0.989; *p* = 0.021). In the remaining quartiles, number of episodes did not moderate the effects of alcohol or smoking (*p*-values of >0.05).

## Discussion

This paper aimed to examine whether the number of previous mood episodes moderates the association between alcohol and smoking on one hand, and the number of depressive, hypomanic and manic days on the other hand, in a one year follow up study of daily life chart scores. First, the overall analyses revealed that an increase in average tobacco consumption per day in a whole year increased the number of depressive days in that year. By contrast, the number of hypomanic and manic days showed no relationship with smoking in the overall analysis, and alcohol consumption was neither related to the number of depressive, hypomanic or manic days. Second, the interaction analyses showed that the number of previous mood episodes moderated the associations between both alcohol use and smoking, and mood. Our most robust finding was that in those with <7 mood episodes (first quartile of number of episodes), one unit increase in average alcohol consumption per day resulted in an adverse and large increase in the number of hypomanic and manic days in that year. By contrast, although to a lesser extent, a decrease in the number of hypomanic and manic days per increased unit of alcohol consumption was found in those subjects with 7 to 13 previous mood episodes (second quartile). A decreased number of hypomanic and manic days was also found per increased unit of tobacco consumption in subjects with 14 to 44 previous mood episodes (third quartile).

Our finding that alcohol is adversely associated with the number of hypomanic and manic days in those with relatively few episodes (first quartile) corroborates our initial hypothesis. We hypothesized that the relationship between alcohol use and smoking on the one hand, and mood on the other are most pronounced in those subjects who have experienced fewer compared to multiple lifetime mood episodes, which is based on Post et al.’s kindling theory [20–22] that states that the impact of exogenous stressors progressively declines with more mood episodes. Likewise, Kendler et al. showed that up to the first nine episodes are linked to stressors and less thereafter [22], although the primary outcome in their analysis was an affective episode, not number of days ill. Thus, the “window of vulnerability” to the adverse relationship between alcohol use and the manic polarity may be comparable to that of other stressors, which is in line with the kindling hypothesis [20–22].

Our finding that this adverse and strong relationship exclusively occurred in the first quartile of mood episodes may be explained by a relatively intact responsiveness to the excitatory, stimulating and destabilizing effects of alcohol in those who experienced only a few previous mood episodes. Intact responsiveness to exogenous effects was also found to CBT effects in those with up to twelve mood episodes [23] and to pharmacotherapeutic effects in those with up to ten episodes [24, 25]; not in those with more episodes. Conversely, an increased number of (hypo) manic days may induce a higher alcohol consumption in the first quartile. Increased alcohol consumption may be mediated by the increased impulsivity and/or excessive involvement in pleasurable activities pertinent to (hypo) mania [27].

In contrast to this increased number of (hypo) manic days per unit alcohol use in the first quartile, we found that – to a lesser extent - the number of hypomanic and manic days decreased per unit alcohol in those subjects in the second quartile. An explanation might be that a decreased number of (hypo) manic days could lead to an increased alcohol consumption in the second quartile of more experienced patients, in an unsuccessful attempt to self-medicate [38] and induce an elevated mood.

Likewise, with each unit increase in tobacco consumption, we also found a modestly decreased number of hypomanic and manic days in those subjects in the third quartile of mood episodes. An explanation may be that, akin to the use of alcohol in the second quartile, a decreased number of (hypo) manic days leads to increased smoking in order to self-medicate [38] and induce positive feelings [27]. Furthermore, smokers may attempt to compensate for the cognitive disturbances [39] that are more frequent among those with more progressed BD [40]. Altogether, the modest effects on (hypo) mania later in the course of illness need further exploration.

In our sample, an increase in average tobacco consumption was independently associated with an increase in the number of depressive days in the whole year. A growing body of literature supports an adverse relationship between smoking and course in BD [11, 12, 41], and the risk of depressive morbidity among smokers is also demonstrated in psychiatric disorders closely related to the bipolar spectrum [42]. Likewise, more severe levels of smoking predicted the onset of major depression [36–39] in well-designed studies that included large samples, adjusted for confounding, and attempted to exclude shared vulnerability factors [43–46]. Alternatively, greater depressive morbidity may cause increased smoking as a mode of self-medication [38], or depression may reduce the success of smoking cessation [6].

Although the relation between smoking and mood is likely to be multifactorial and multidirectional [7, 14], unidirectional effects of smoking on depressive days in BD may be mediated by several neurochemical mechanisms. First, nicotine is a nicotinic cholinergic receptor agonist that affects acetylcholine, catecholamine and dopamine systems [40, 42, 47], and these systems play an etiologic role in depression [48] including bipolar depression [49]. Second, polycyclic aromatic hydrocarbons (PACs) and other compounds in cigarette smoke induce oxidative stress, and elevated markers of oxidative stress are positively correlated with the severity of depression [50]. Third, PACs in cigarettes can interfere with the efficacy of pharmacotherapy in BD [25, 45, 51] by lowering serum levels of several psychotropic drugs through hepatic enzyme induction [52].

Contrary to our initial hypothesis we did not find any overall association between alcohol and depression. The absence of such an overall effect is in agreement with some previous studies [13, 18]. The explanation suggested by Van Zaane et al. [17], that alcohol may have an effect on mood only in the early course of BD, is supported by our finding that the most robust relationship between alcohol use and (hypo)mania occurred exclusively in those with few previous mood episodes and not in the overall sample.

Strengths of our study are the daily, prospective and monthly double-checked measurements, and a detailed assessment of baseline alcohol use and smoking levels. Limitations are the representativeness of our sample, as all participants were Dutch [53] and treatment adherent [17]. In addition, one could argue that our analyses should have been adjusted for percentage, type or number of medication used, however we considered this of no additional value due to the large percentage of 92.5% of medication use in our sample, no variation in medication adherence, and the complex nature of response to medication in BD [54]. Furthermore, we only considered current substance use and did not take into account the possible lifetime-accumulated effects of substance use on sensitization processes [20, 50, 55]. For future research, it would be informative to investigate short-term co-variation of daily mood scores during a year with daily alcohol and drugs consumption and other behavioral information that can be collected with life charts, such as hours of sleep and life events.

## Conclusion

Our study indicates that the number of previous mood episodes moderates the association between alcohol use and smoking, and mood. Although these findings should be interpreted with caution, the adverse and strong relationship between alcohol and (hypo)mania in those with few lifetime mood episodes is in agreement with the kindling hypothesis. This may suggest that particularly patients early in the course of bipolar disorders are vulnerable to the adverse interaction between alcohol and (hypo)mania. The opposite and smaller associations between alcohol use and (hypo)mania, and between smoking and (hypo)mania in those with more previous mood episodes need further replication and exploration of possible underlying mechanisms. The adverse overall relationship between smoking and depression, regardless of the direction of the effect, deserves major attention from clinicians.
